# A diagnosis based on the electrocardiogram before laboratory results were available

**DOI:** 10.1007/s12471-015-0712-7

**Published:** 2015-05-13

**Authors:** S.L. Gerritse, E.H.C.C. Janssen

**Affiliations:** 1 Amphia Hospital, Molengracht 21, 4818 CK Breda, The Netherlands; 2 Erasmus MC, Rotterdam, The Netherlands

**Fig. 1:**
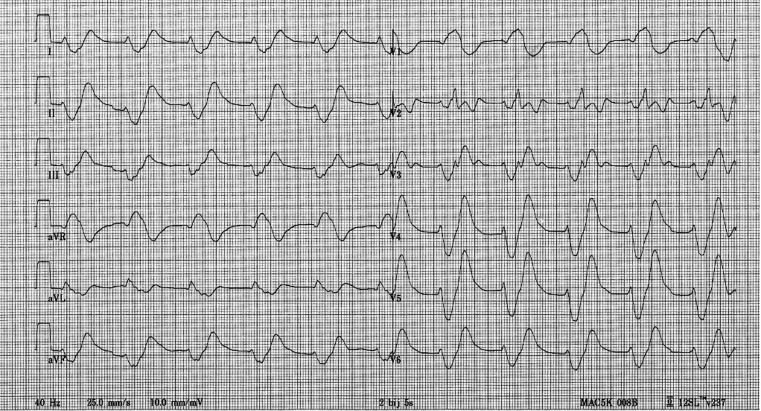
Electrocardiogram on admission

A 81-year-old male patient is brought in by ambulance with persistent diarrhoea and subacute symptoms of lethargy, weakness of the leg muscles and malaise. He is taking perindopril 4 mg twice a day. On physical examination you find a decreased level of consciousness and a (pre-existing) systolic murmur, fitting his mitral valve insufficiency. His electrocardiogram on admission is enclosed in Fig. 1. What would you expect to find in your laboratory results?

You will find the answer elsewhere in this issue.

